# A humanized CD3ε-knock-in mouse model for pre-clinical testing of anti-human CD3 therapy

**DOI:** 10.1371/journal.pone.0245917

**Published:** 2021-02-17

**Authors:** Joel Crespo, Yi Ting Koh, Ningjie Hu, Paul A. Moore, Ezio Bonvini, Andrew L. Glasebrook, Andrea P. Martin, Robert J. Benschop

**Affiliations:** 1 Immunology Research, Lilly Research Laboratories, Eli Lilly and Company, Indianapolis, IN, United States of America; 2 MacroGenics, Rockville, MD, United States of America; Institut National de la Santeet de la Recherche Medicale (INSERM), FRANCE

## Abstract

Pre-clinical murine models are critical for translating drug candidates from the bench to the bedside. There is interest in better understanding how anti-human CD3 therapy works based on recent longitudinal studies of short-term administration. Although several models have been created in this pursuit, each have their own advantages and disadvantages in Type-1 diabetes. In this study, we report a murine genetic knock-in model which expresses both a murine and a humanized-CD3ε-exon, rendering it sensitive to manipulation with anti-human CD3. These huCD3ε^HET^ mice are viable and display no gross abnormalities. Specifically, thymocyte development and T cell peripheral homeostasis is unaffected. We tested immune functionality of these mice by immunizing them with T cell-dependent antigens and no differences in antibody titers compared to wild type mice were recorded. Finally, we performed a graft-vs-host disease model that is driven by effector T cell responses and observed a wasting disease upon transfer of huCD3ε^HET^ T cells. Our results show a viable humanized CD3 murine model that develops normally, is functionally engaged by anti-human CD3 and can instruct on pre-clinical tests of anti-human CD3 antibodies.

## Introduction

Monoclonal antibodies are versatile biologic agents known to improve outcomes in autoimmune, transplant rejection and malignant diseases. These may work in a variety of ways, for example by 1) dampening inflammatory immune or cellular responses [1–4], 2) activating the immune response [5–7], or 3) inducing a state of immune tolerance [8–10]. Given the diversity of these indications, there is considerable interest in being able to test potential and actual human therapeutic antibodies in pre-clinical models that mimic what is observed in the clinic and may therefore instruct on the mechanism of action.

Monoclonal antibodies to CD3 have been used in the clinic to help in organ transplantation and treat autoimmune diseases with varying degrees of success. Patients have received anti-CD3 therapy to suppress acute graft-rejection or acute renal failure following kidney transplantation and ensure long-term survival of the organ through the short-term depletion of graft-targeting T cells [1, 2]. Recently diagnosed Type-1 diabetes (T1D) patients have also received anti-CD3 therapy. Anti-CD3 therapy in recent-onset T1D patients led to short-term stabilization of C-Peptide levels, similar to those observed in healthy controls [11, 12]. Interestingly, long-term responders to anti-CD3-therapy showed an increase in co-inhibitory receptor co-expression by T cells reminiscent of that observed by exhausted or anergic T cells of cancer patients [13, 14]. The biology underlying these treatments is complex and not completely understood. Therefore, having suitable preclinical models may help to further our understanding towards mechanisms.

A major hurdle for understanding the mechanism through which anti-human CD3 therapy works is that these antibodies are species-specific and do not cross-react with the murine targets. Several approaches have been developed to work through these including the development of humanized-mouse models with transgenic expression of human CD3 components which respond to anti-human CD3 antibodies or the engraftment of the human hematopoietic system into immune-deficient mice, though each of these approaches have specific limitations. Several groups have introduced the human CD3ε gene into either the non-obese diabetic (NOD) or C57BL/6 mouse strains with different degrees of success [15–17]. CD3ε is commonly used since most anti-human CD3 antibodies recognize CD3ε epitopes and provides a structural and signaling role in the TCR-CD3 complex. It was shown that genetic knockout leads to blockade in thymocyte development and therefore peripheral T cells. Replacement of the murine CD3ε seems an attractive method since it would allow for normal development of the murine immune system. However, it was first shown that human CD3ε introduction affected normal thymocyte development and peripheral T cell numbers. The introduction was carried out by injecting fertilized eggs with a plasmid containing the human CD3ε gene which resulted in transgenic mouse lines with varied transgene copy numbers. Those mouse lines with higher copy numbers showed lower peripheral T cell numbers, showing the importance of the murine CD3ε protein in the structure and function of the TCR-CD3 complex. Ueda, et al. took a different approach and introduced humanized versions of all CD3 complex components epsilon (ε), delta (δ), and gamma (γ) into mice. T cells from *CD3 EDG (*εδγ) mice were shown to proliferate and release cytokines in response to anti-human CD3 stimulation. However, *CD3 EDG* mice also show discreet changes in CD3 frequency, CD4:CD8 ratios, and changes in their immunoglobulin production following immunization due to changes in T cell function *in vivo* [17]. NOD-huCD3ε mice were also described [16]. NOD-huCD3ε mice were similar in T cell phenotype to their wild-type (WT) counterparts and responded to anti-human CD3 stimulation. However, two disadvantages are associated with this model, 1) this model will always develop diabetes, which may impede the study of other immunological diseases, and 2) the model’s background is locked into the NOD mouse strain preventing the study of several experimental models in other mouse strains.

The alternative approach is the engraftment of the human immune system into immune-deficient mice which is also imperfect as 1) several immune cell subsets do not develop and mature in the periphery due to a lack of human cytokines which can result in impaired immunity, 2) major and minor histocompatibility complex (MHC) mismatches which result in unfit thymocyte development, and 3) donor-to-donor variances in engraftment in mice and MHC matching [18, 19]. Several reports have tried to address these by overexpressing human cytokines to aid in the development of immune cell subsets, including MHC matched to the human donor with varying degrees of success, or co-transplantation of human CD34^+^ fetal liver cells and human fetal thymic tissue [20–25]. However, full recapitulation of the human immune system may ultimately require the compounded effects of each of these genetic manipulations on the mouse and would further require tailoring of the specific human donor MHC which makes this model difficult to work with. As each of these models have their own caveats, we aimed to develop our own mouse model wherein anti-human CD3 antibodies could be studied.

This study describes a novel murine model in which a single murine CD3ε-exon was replaced with a humanized CD3ε-exon containing the epitope for anti-human CD3 OKT3 antibody. We found that huCD3ε mice homozygous for human CD3ε (huCD3ε^HOM^), showed decreased peripheral T cell numbers with abnormal CD4:CD8 T cell ratio and phenotype when compared to WT mice. These changes were traced back to impaired early thymocyte selection, in fact huCD3ε^HOM^ thymi displayed gross abnormalities and loss of morphology and cellularity. In contrast, we observed co-expression of both mouse and human CD3ε in T cells from CD3ε^-HET^ animals and these displayed normal T cell development and peripheral T cell compartment compared to WT mice. We therefore continued our studies in huCD3ε^HET^ to determine whether these mice could be used to study the biology associated with anti-human CD3 antibodies. Furthermore, huCD3ε^HET^ mice were able to mount effective immune responses using two models which tested T cell-dependent responses *in vivo*, showing that replacement one murine CD3ε-exon with the humanized CD3ε-exon did not impair these responses. We also demonstrated that huCD3ε^HET^ T cells responded to anti-human CD3 stimulation *in vitro* and *in vivo*. Overall, we show that this new mouse model huCD3ε^HET^ mice in the C57BL/6 background allows for study of anti-human CD3 biology and may address several limitations that previous models have been unable to.

## Materials and methods

### Mice

HuCD3ε^HET^ and huCD3ε^HOM^ mice in the C57BL/6 background were generated at Artemis, a subsidiary of Taconic Biosciences using conventional techniques and housed at Taconic facilities. All experiments were approved and carried out following the Eli Lilly and Company Institutional Animal Care and Use Committee research guidelines. Specifically, all animals used in this study were cared for according to the guidelines in the Guide for the Care and Use of Laboratory Animals. Lilly Research Laboratories are AAALAC-accredited facilities. All animals were placed on a standard 12 h light: 12 h dark cycle. Animals were housed in groups of 4–5 per cage with free access to water and food. The room temperature setpoint was 72°F +/- 2°F and humidity range was between 30–70%. Mice were euthanized by carbon dioxide asphyxiation followed by an approved secondary method including cervical dislocation or exsanguination. B6D2 and C57BL/6 WT mice were obtained from Charles River or Taconic Biosciences. Littermate controls are used in select experiments.

### Mouse samples and cell isolation

Cells were collected from various organs (e.g., bone marrow, spleen, cervical and mesenteric lymph nodes or peripheral blood) and processed into single-cell suspensions. Blood was obtained by heart puncture and cell number was determined through complete blood count using Hemavet (Drew Scientific Inc). After lysis of red blood cells, cells were resuspended in culture medium (RPMI1640 with L-glutamine containing 10% FBS, 1% non-essential amino acids, 1 mM sodium pyruvate, 100 U/mL penicillin, 100 μg/mL streptomycin, Gibco). Cells were either cultured or stained for multiple-color flow cytometry as described below.

### Reagents

Antibodies used for flow cytometry were CD5 (55–7.3) (eBiosciences/ThermoFischer), CD8 (53–6.7), CD4 (RM4-5), CD44 (IM7), CD62L (MEL-14), and Annexin-V (BD Biosciences), CD3 (OKT3), CD3 (145-2C11), CD69 (H1.2F3) (BioLegend). 7-AAD, propidium iodide (BD Biosciences) or zombie aqua (BioLegend) were used for viability staining. Anti-mouse TCR-Vβ screening panel (BD Biosciences). Antibodies used for *in vitro* functional studies or *in vivo* treatment were anti-human CD3 (Clone OKT3), anti-mouse CD3ε (Clone 145-2C11) (BD Biosciences or generated in-house) and anti-mouse TCRβ (H57-597 from eBiosciences/ThermoFischer).

### *In vitro* T cell activation cell cultures

Wells were coated overnight at 4°C with anti-human CD3 (OKT3, 1.0 μg/mL), anti-mouse CD3 (145-2C11, 1.0 μg/mL), or anti-mouse TCRβ (1.0 μg/mL), and their respective isotype controls (mouse IgG2a or Armenian hamster IgG). Wells were washed with PBS prior to experiment start. Splenocytes from huCD3ε^HET^ or WT (1.0 × 10^6^ cells/mL) were seeded in 12-well flat-bottom tissue culture plates in culture media. Cells were incubated for 24-36hrs at 37°C and 5%CO_2_. Cells were then stained for multiple-color flow cytometry as described below.

### Flow cytometry, surface and intracellular antigen detection

Freshly obtained single-cells from tissues or *in vitro* cultured splenocytes were stained in Annexin-V staining buffer (BD Biosciences) using fluorophore-conjugated antibodies against extracellular antigens followed by 7-AAD or PI staining, then read on the flow cytometer. Alternatively, cells were stained for extracellular antigens with fluorophore-conjugated antibodies in PBS with 2% fetal bovine serum for 30 mins on ice. 7-AAD or PI was added to cells directly before acquisition on flow cytometer. Samples were analyzed on either FC500 (Coulter), Fortessa (BD Biosciences), or ZE5 Cell Analyzer (BioRad). Flow cytometry data was analyzed using FCS Express (De Novo Software) and FlowJo (BD Biosciences) software.

### Histology

Tissue was dissected, fixed in 10% phosphate-buffered formalin and then processed for paraffin sections. Five-micrometer sections were stained with hematoxylin and eosin.

### *In vivo* T cell depletion experiments

6–10 weeks-old huCD3ε^HET^ or WT mice were injected *i*.*p*. with anti-human CD3 (500 μg/mouse), anti-mouse CD3 (500 μg/mouse), or vehicle control. Blood was collected 24hrs post-injection and cellularity was measured through flow cytometry.

### Graft vs. host disease model

2.5 million huCD3ε^HET^ or WT T cell-depleted bone marrow were mixed with 5 million unfractionated matched-splenocytes and injected intravenously into 8–9 weeks-old B6D2 recipients irradiated at 800 rads. Sulfamethoxazole trimethoprim oral suspension (240 mg/5 mL/bottle) was added to the animal’s drinking water throughout the experiment. Mice were weighed daily and euthanized upon reaching 80% of initial body weight.

### Immunization

Female (3–4 months-old) mice were immunized i.p. with 2 μg NP-CGG (Biosearch Technologies) or 50 μg ovalbumin (OVA, Sigma-Aldrich, St. Louis, MO) precipitated in alum (Pierce, Rockford, IL). At day 14 mice were injected i.p. with 2 μg NP-CGG or 50 μg OVA in sterile PBS. Serum was collected at 10 and 21 days to measure the primary and secondary immune response, respectively. Anti-OVA and anti-NP-CGG antibody titers were measured using a proprietary ELISA.

### Statistical analysis

All data were analyzed using GraphPad Prism for Windows ver. 8.2.1. Analysis that consider all three genetic backgrounds were tested using one-way Anova test followed by Tukey’s multiple comparisons test. Mann-Whitney two-tailed test was used to test for significance where two genetic backgrounds were compared. Data are expressed as mean ±SEM and considered significant at p<0.05.

## Results

### Generation of huCD3ε-knock-in mice

**Fig 1:**
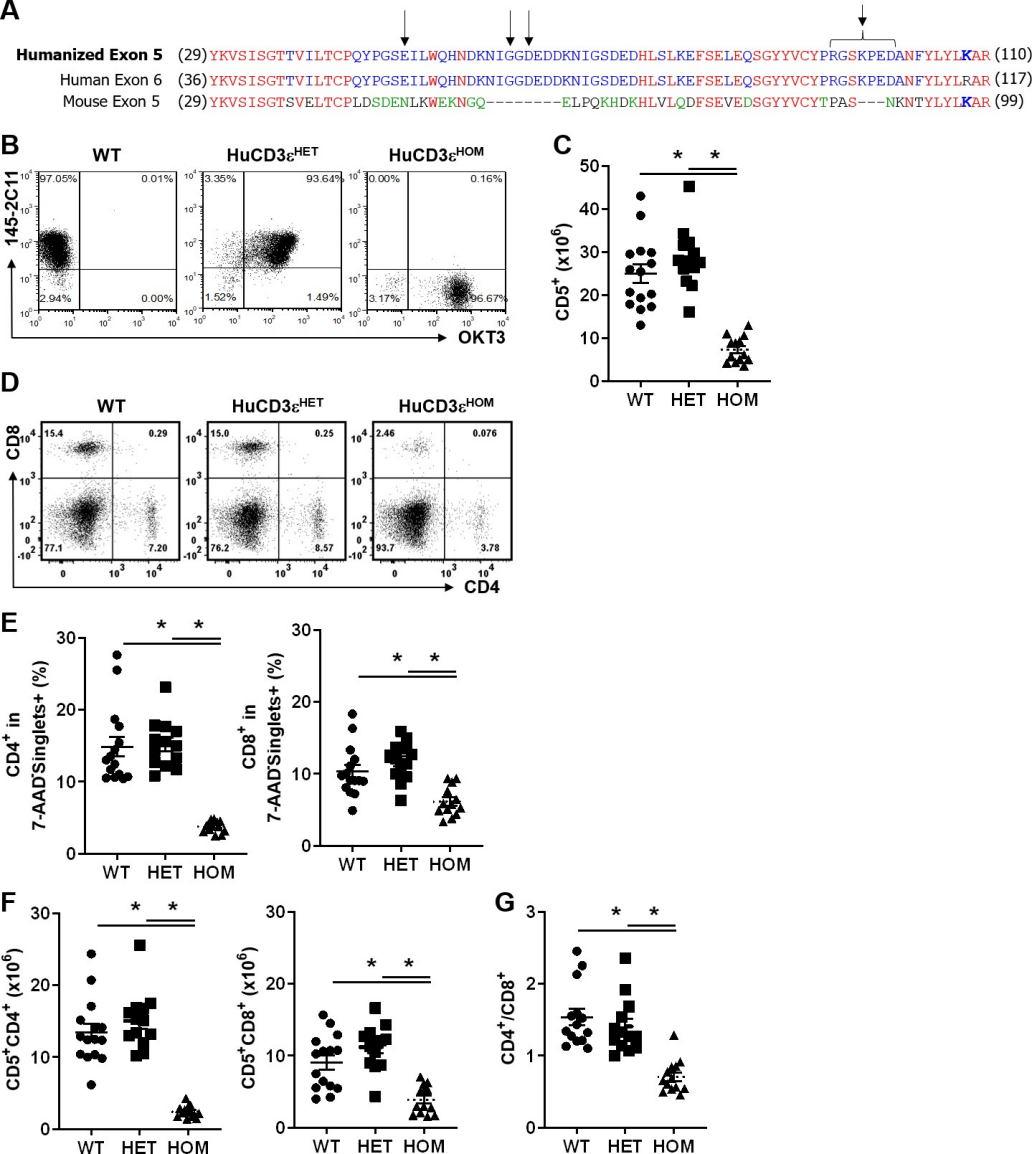
T cells from huCD3ε^HET^ and huCD3ε^HOM^ mice express OKT3 binding site. (A) Amino acid alignment of corresponding human and mouse CD3e exons. Arrows indicate residues that have been shown to be necessary for OKT3 binding [17]. Mouse exon 5 was replaced with a humanized exon 5 (top line) to generate huCD3ε^HET^ or huCD3ε^HOM^ mice. (B) Representative flow cytometry plots of mouse splenocytes (gated on scatter profile, PI exclusion, and CD5) using antibodies specific for human (OKT3) or mouse (145-2C11) CD3ε, comparing WT, huCD3ε^HET^, and huCD3ε^HOM^ mice. (C) Absolute T cell numbers across each genotype. n = 5–13, p<0.05. (D-F) Representative flow cytometry plots showing the frequency of CD4^+^ and CD8^+^ T cells (D), summarized frequencies (E), and absolute cell numbers (F) obtained from spleens of 6-8-week old mice from each genotype. Cells are gated on scatter profile and PI exclusion. (G) CD4:CD8 T cell number ratio from spleens. n = 13–15, *p<0.05.

The binding epitope for the OKT3 anti-human CD3ε is encoded by exon 6 (exon 5 is the homologous mouse exon) [26, 27]. We generated CD3ε-knock-in mice, where mouse exon 5 was replaced by the corresponding human exon 6. To preserve the native interaction between CD3ε with CD3δ and CD3γ, the amino acid Arginine at the C terminal end of the human exon 6 was mutated to the mouse amino acid Lysine (R->K; Fig 1A). The humanized exon encodes for 11 additional amino acids compared to mouse exon 5, some of which form the binding epitope for OKT3 [27]. Since the resulting targeting vector contained a puromycin selectable marker flanked by FLP sequences, the puromycin selectable marker was eventually removed by crossing the huCD3ε-carriers onto a FLP-deleter mouse [28]. Founder mice on the C57BL/6 background were selected and bred to generate heterozygous (huCD3ε^HET^) and homozygous (huCD3ε^HOM^) huCD3ε-knock-in mice. Mice were healthy and fertile and had no gross abnormalities. We measured the expression of human and mouse CD3ε on splenic T cells from WT, huCD3ε^HET^ and huCD3ε^HOM^ mice by flow cytometry (Fig 1B and 1C). T cells from huCD3ε^HOM^ mice stained exclusively with OKT3, while T cells from huCD3ε^HET^ mice expressed both human and murine CD3ε on their cell surface (Fig 1B) suggesting the successful integration of the humanized CD3ε exon into the murine CD3 gene. HuCD3ε^HOM^ mice showed an approximate 3-fold reduction in the number of T cells in the spleen compared to WT or huCD3ε^HET^ mice (WT:24.95x10^6^ ± 2.16, huCD3ε^HET^:28.48x10^6^ ± 1.91, huCD3ε^HOM^:7.31x10^6^ ± 0.85) (Fig 1C). HuCD3ε^HET^ mice showed similar relative and absolute numbers of CD4 and CD8 subpopulations compared to WT mice (Absolute CD4^+^ T cell numbers: WT:13.48x10^6^ ± 1.17, huCD3ε^HET^:15.02x10^6^ ± 1.10, huCD3ε^HOM^:2.39x10^6^ ± 0.22. Frequency of CD4+ T cells: WT:14.87% ± 1.40, huCD3ε^HET^:15.16% ± 0.91, huCD3ε^HOM^:3.73% ± 0.22. Absolute CD8^+^ T cell numbers: WT:9.08x10^6^ ± 1.00, huCD3ε^HET^:11.18x10^6^ ± 0.90, huCD3ε^HOM^:3.86x10^6^ ± 0.55. Frequency of CD8^+^ T cells: WT:10.30% ± 0.90, huCD3ε^HET^:11.73% ± 0.74, huCD3ε^HOM^:6.14% ± 0.57) (Fig 1D–1F). However, huCD3ε^HOM^ had significantly lower CD4 and CD8 T cell numbers in the spleen and an altered CD4:CD8 T cell number ratio (WT:1.54 ± 0.12, huCD3ε^HET^:1.41 ± 0.11, huCD3ε^HOM^:0.70 ± 0.06) (Fig 1G).

### Thymocyte development is impaired in huCD3ε^HOM^ mice but not in huCD3ε^HET^

**Fig 2:**
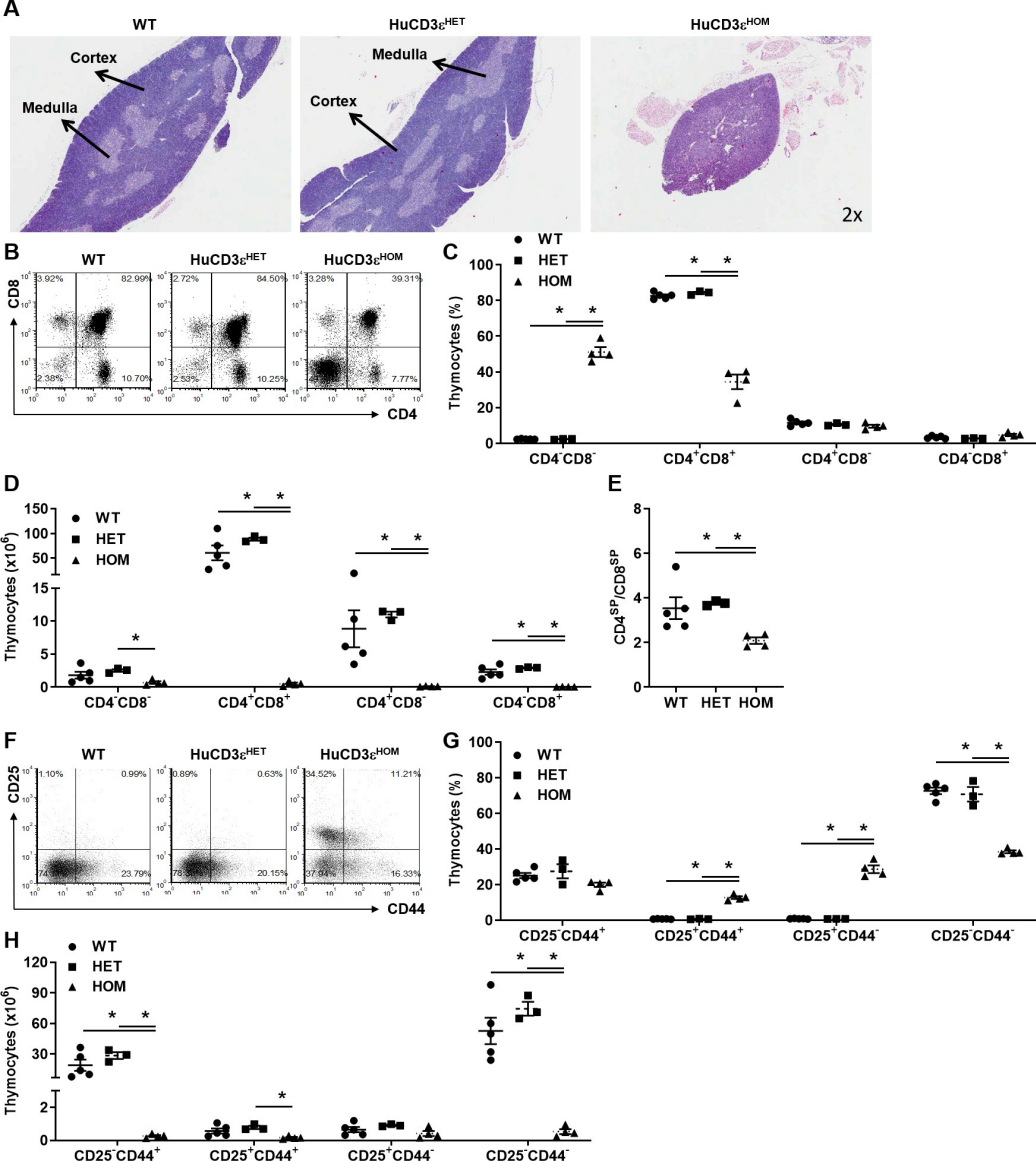
HuCD3ε^HOM^, but not huCD3ε^HET^ mice, show impaired thymocyte development. (A) Representative histological staining (H&E) of thymi from WT, huCD3ε^HET^, and huCD3ε^HOM^ mice. (B) Flow cytometry plots of murine thymocytes (gated on scatter profile and B220^-^) comparing CD8^+^ x CD4^+^ thymocytes from each genotype. (C-D) Graphs showing frequencies (C) and absolute cell numbers (D) from (B). (E) Cell number ratio of CD4^+^CD8^-^ to CD4^-^CD8^+^ thymocytes observed from each genotype. (F) Flow cytometry plots of murine thymocytes comparing CD25^+^ x CD44^+^ thymocytes. Cells are gated on scatter profile and B220^-^. (G-H) Graphs showing frequencies (G) and absolute cell numbers (H) from (F). n = 3–5, *p<0.05.

To understand the probable cause for the significantly reduced T cell numbers in huCD3e^HOM^ mice, we first investigated thymus morphology and cellularity. Thymus from HuCD3ε^HOM^ mice were significantly smaller than those from WT or huCD3ε^HET^ (Fig 2A). H&E staining showed dramatic loss of cellularity and a significant reduction of the medulla and cortex thymic epithelial spaces in huCD3ε^HOM^, compared to WT and huCD3ε^HET^. We hypothesized that loss of thymus morphology in huCD3ε^HOM^ mice would be accompanied with impaired thymocyte development therefore, we measured the absolute numbers and frequency of different thymocyte developmental stages by flow cytometry. We first looked at the distribution of thymocytes based on the surface expression of CD4 and/or CD8. HuCD3ε^HOM^ thymocytes were predominantly found in the CD4^-^CD8^-^ double negative (DN) stage, where the frequency of DN cells was significantly higher and other stages (CD4^+^CD8^+^, CD4^+^CD8^-^ and CD4^-^CD8^+^) decreased, when compared to WT and huCD3ε^HET^ (WT CD4^-^CD8^-^:2.40% ± 0.77, CD4^+^CD8^+^:82.65% ± 0.81, CD4^+^CD8^-^:11.54% ± 0.75, CD4^-^CD8^+^:3.40% ± 0.34. HuCD3ε^HET^ CD4^-^CD8^-^:2.40% ± 0.15, CD4^+^CD8^+^:84.28% ± 0.61, CD4^+^CD8^-^:10.53% ± 0.43, CD4^-^CD8^+^:2.79% ± 0.07. HuCD3ε^HOM^ CD4^-^CD8^-^:51.16% ± 2.81, CD4^+^CD8^+^:34.45% ± 4.06, CD4^+^CD8^-^:9.65% ± 0.85, CD4^-^CD8^+^:4.74% ± 0.67) (Fig 2B and 2C). In line with having decreased thymic cellularity, the absolute huCD3ε^HOM^ thymocyte number was decreased across all developmental stages, compared to both WT and huCD3ε^HET^ mice (WT CD4^-^CD8^-^:1.81x10^6^ ± 0.52, CD4^+^CD8^+^:60.52x10^6^ ± 15.00, CD4^+^CD8^-^:8.85x10^6^ ± 2.80, CD4^-^CD8^+^:2.30x10^6^ ± 0.41. HuCD3ε^HET^ CD4^-^CD8^-^:2.52x10^6^ ± 0.20, CD4^+^CD8^+^:88.33x10^6^ ± 3.11, CD4^+^CD8^-^:11.02x10^6^ ± 0.44, CD4^-^CD8^+^:2.92x10^6^ ± 0.08. HuCD3ε^HOM^ CD4^-^CD8^-^:0.69x10^6^ ± 0.19, CD4^+^CD8^+^:0.51x10^6^ ± 0.18, CD4^+^CD8^-^:0.12x10^6^ ± 0.03, CD4^-^CD8^+^:0.06x10^6^ ± 0.01) (Fig 2D). We also noted that the CD4^+^ to CD8^+^ ratio in the thymus from huCD3ε^HOM^ mice was skewed towards CD8^+^ thymocytes, suggesting that CD4^+^ T cell development was more impacted than the development of CD8^+^ T cells (WT:3.55 ± 0.49, huCD3ε^HET^:3.77 ± 0.06, huCD3ε^HOM^:2.09 ± 0.14) (Fig 2E). These observations suggest thymocyte development is impaired prior to the DN stage of thymocyte development in huCD3ε^HOM^ mice.

Prior to the thymocyte stages marked by CD4 and/or CD8 surface expression, thymocytes are tracked based on the expression of CD25 and CD44 in the following order: DN1-CD25^-^CD44^+^, DN2-CD25^+^CD44^+^, DN3-CD25^+^CD44^-^, DN4-CD25^-^CD44^-^ [29]. We used these markers to understand in which stage huCD3ε^HOM^ thymocytes are impaired. Overall, all CD4^-^CD8^-^ thymocyte cell subsets were significantly decreased in huCD3ε^HOM^ mice compared to WT and huCD3ε^HET^ mice (Fig 2F and 2G). Specifically, we observed an increase in the frequency of huCD3ε^HOM^ thymocyte subsets DN2,3 with an accompanying decrease in DN1,4 cells, as compared to thymocyte subsets in WT and huCD3ε^HET^ mice (WT CD25^-^CD44^+^:25.16% ± 1.48, CD25^+^CD44^+^:0.79% ± 0.07, CD25^+^CD44^-^:0.91% ± 0.06, CD25^-^CD44^-^:72.72% ± 1.84. HuCD3ε^HET^ CD25^-^CD44^+^:27.56% ± 3.97, CD25^+^CD44^+^:0.76% ± 0.11, CD25^+^CD44^-^:0.87% ± 0.02, CD25^-^CD44^-^:70.81% ± 4.07. HuCD3ε^HOM^ CD25^-^CD44^+^:19.99% ± 1.23, CD25^+^CD44^+^:12.79% ± 0.78, CD25^+^CD44^-^:28.74% ± 2.16, CD25^-^CD44^-^:38.50% ± 0.76). Further, the absolute huCD3ε^HOM^ thymocyte numbers were significantly lower in each cell subset except for DN3-CD25^+^CD44^-^ cells, as compared to WT and huCD3ε^HET^ (WT CD25^-^CD44^+^:19.13x10^6^ ± 5.50, CD25^+^CD44^+^:0.57x10^6^ ± 0.15, CD25^+^CD44^-^:0.65x10^6^ ± 0.16, CD25^-^CD44^-^:52.75x10^6^ ± 12.91. HuCD3ε^HET^ CD25^-^CD44^+^:28.62x10^6^ ± 3.35, CD25^+^CD44^+^:0.79x10^6^ ± 0.10, CD25^+^CD44^-^:0.91x10^6^ ± 0.04, CD25^-^CD44^-^:74.47x10^6^ ± 6.72. HuCD3ε^HOM^ CD25^-^CD44^+^:0.27x10^6^ ± 0.06, CD25^+^CD44^+^:0.17x10^6^ ± 0.40, CD25^+^CD44^-^:0.42x10^6^ ± 0.15, CD25^-^CD44^-^:0.53x10^6^ ± 0.15) (Fig 2H).

Taken together, huCD3ε^HOM^ thymocyte development is impaired early at the DN1-CD25^-^CD44^+^ stage whereas thymocytes in huCD3ε^HET^ mice showed no differences compared to WT. The observed decreased huCD3ε^HOM^ T cell numbers in the periphery is explained by abnormal thymocyte development. HuCD3ε^HET^ thymocytes did not show any gross abnormalities throughout their stages of development as they were comparable to thymocytes from WT mice. This shows dual expression of the murine and humanized CD3ε protein does not impede thymocyte development.

### HuCD3ε^HET^ mice show normal peripheral T cell phenotype

**Fig 3:**
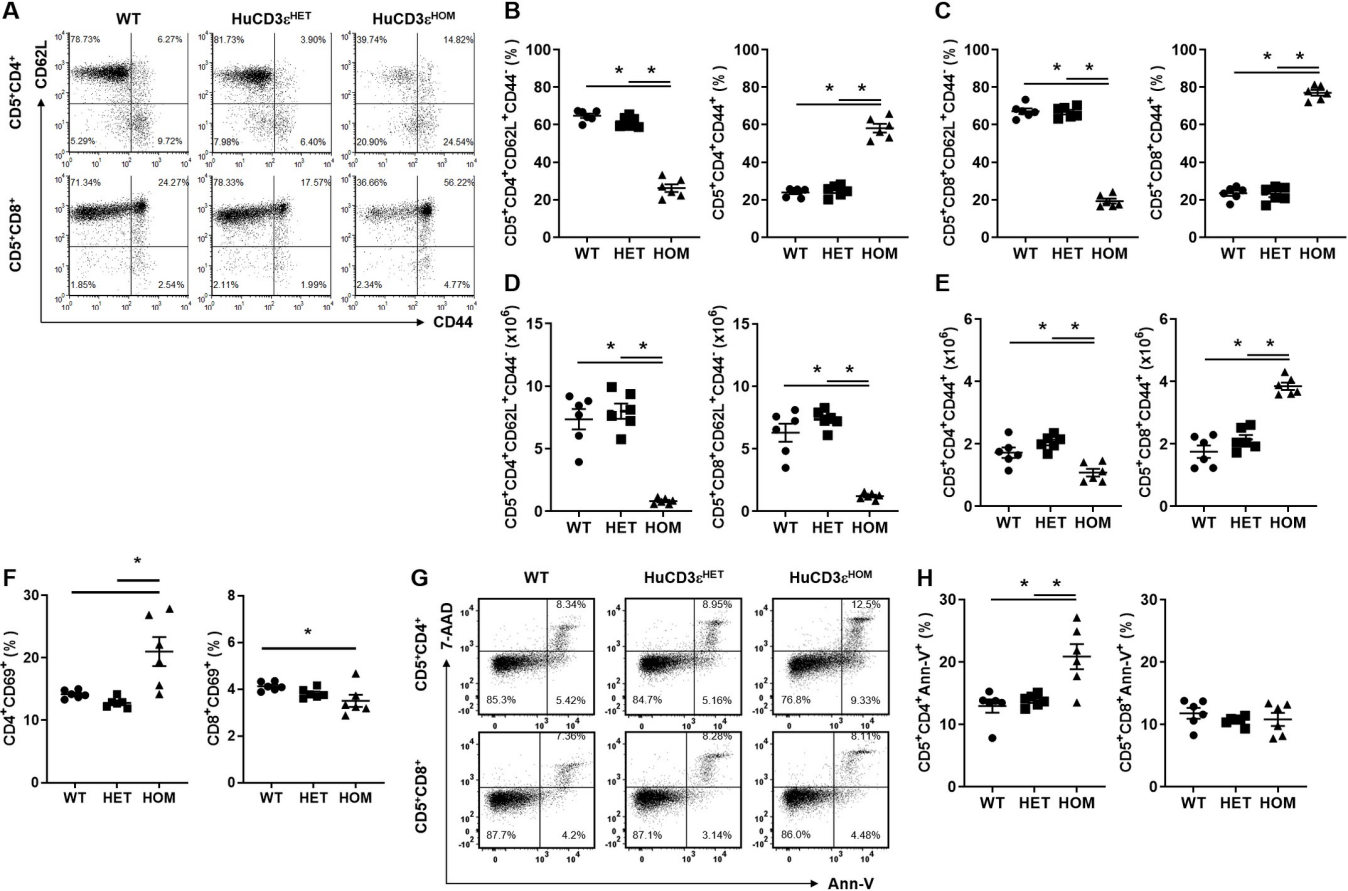
HuCD3ε^HET^ peripheral T cells show normal phenotype. (A) Representative flow cytometry plots of mouse splenocytes comparing CD62L^+^ x CD44^+^ expression on CD5^+^CD4^+^ (top row) or CD5^+^CD8^+^ (bottom row) WT, huCD3ε^HET^ and huCD3ε^HOM^ T cells. (B-E) Graphs summarizing the frequencies and absolute cell numbers of CD62L^+^CD44^-^ CD4^+^ or CD8^+^ T cells (B, D) and CD44^+^ CD4^+^ or CD8^+^ T cells (C, E) from each genetic background. (F) Graphical summary of CD69 expression on CD4^+^ or CD8^+^ T cells from naïve mice from each genotype. (G) Representative flow cytometry plots of apoptosis as measured by Ann-V x 7-AAD in fresh T cells of naïve mice. (H) Graphical summary of (G). n = 5–6, *p<0.05.

It has previously been reported that replacement of murine-to-human CD3 gene in mice leads to aberrations in T cell phenotype, frequency and absolute numbers [15, 17]. To understand if our CD3ε-knock-in mice showed alterations of peripheral T cells, we studied the survival, phenotype, frequency and, numbers of T cells in spleens of mice from each genotype. We found neither the frequency nor absolute numbers of T cells from huCD3ε^HET^ mice showed any changes in the naïve (CD62L^+^CD44^-^) or memory (CD44^+^) compartments, compared to WT mice (Fig 3A–3E). In contrast, huCD3ε^HOM^ showed a decrease in naïve T cell frequency, together with an increase in memory T cell frequency in both the CD4^+^ and CD8^+^ compartments compared to WT mice (frequency of CD4^+^CD62L^+^CD44^-^ T cells WT:64.75% ± 1.20, huCD3ε^HET^:61.6% ± 1.15, huCD3ε^HOM^:26.20% ± 2.04. Frequency of CD4^+^CD44^+^ T cells WT:23.88% ± 0.93, huCD3ε^HET^:24.75% ± 1.20, huCD3ε^HOM^:58.13% ± 2.31. Frequency of CD8^+^CD62L^+^CD44^-^ T cells WT:67.23% ± 1.49, huCD3ε^HET^:66.72% ± 1.20, huCD3ε^HOM^:19.30% ± 1.30. Frequency of CD8^+^CD44^+^ T cells WT:23.47% ± 1.41, huCD3ε^HET^:22.97% ± 1.61, huCD3ε^HOM^:77.07% ± 1.54) (Fig 3A–3C). Further, absolute CD4^+^ T cell numbers in huCD3ε^HOM^ mice were significantly decreased for both naïve and memory cell subsets, compared to WT mice (absolute cell number of CD4^+^CD62L^+^CD44^-^ T cells WT:7.36x10^6^ ± 0.82, huCD3ε^HET^:8.01x10^6^ ± 0.62, huCD3ε^HOM^:0.83x10^6^ ± 0.10. Absolute cell number of CD4^+^CD44^+^ T cells WT:1.72x10^6^ ± 0.17, huCD3ε^HET^:2.05x10^6^ ± 0.09, huCD3ε^HOM^:1.08x10^6^ ± 0.12. Absolute cell number of CD8^+^CD62L^+^CD44^-^ T cells WT:6.30x10^6^ ± 0.73, huCD3ε^HET^:7.37x10^6^ ± 0.30, huCD3ε^HOM^:1.23x10^6^ ± 0.11. Absolute cell number of CD8^+^CD44^+^ T cells WT:1.75x10^6^ ± 0.20, huCD3ε^HET^:2.15x10^6^ ± 0.14, huCD3ε^HOM^:3.84x10^6^ ± 0.12) (Fig 3D and 3E). However, CD8^+^CD44^+^ T cells in huCD3ε^HOM^ mice were increased as compared to cells from WT mice (Fig 3E).

We also analyzed TCR-Vβ usage by flow cytometry to determine TCR repertoire and found that huCD3ε^HET^ CD8^+^ T cells displayed some small yet significant changes, whilst huCD3ε^HET^ CD4^+^ T cells were identical to WT, (S1A and S1B Fig). Specifically, Vβ3, Vβ5.1/2, and Vβ11 showed a statistically significant decrease (S1A Fig). However, these changes were much more dramatic on huCD3ε^HOM^ T cells as compared to WT where most TCR-Vβ assayed showed an altered frequency in both CD4^+^ and CD8^+^ T cells. This is not surprising due to the impaired thymocyte selection observed above. We questioned whether T cell numbers normalized as a function of age. We found that both CD4^+^ and CD8^+^ T cell numbers normalized in the spleen of huCD3ε^HOM^ mice to those observed in WT mice (S2A and S2B Fig). This is mainly driven by an increase of CD44^+^ memory and concurrent decrease of CD62L^+^CD44^-^ naïve T cells in aged mice. No differences were detected between huCDε3^HET^ and WT aged mice.

We next evaluated the activation state of CD4^+^ and CD8^+^ T cells by measuring the frequency of CD69^+^ cells by flow cytometry. The frequency of CD4^+^CD69^+^ and CD8^+^CD69^+^ huCD3ε^HET^ T cells was comparable to WT whereas huCD3ε^HOM^ CD4^+^CD69^+^ T cells were increased (frequency of CD4^+^CD69^+^ WT:11.06% ± 0.15, huCD3ε^HET^:8.33% ± 0.35, huCD3ε^HOM^:13.00% ± 1.53. Frequency of CD8^+^CD69^+^ WT:3.74% ± 0.20, huCD3ε^HET^:3.01% ± 0.13, huCD3ε^HOM^:3.72% ± 0.19) (Fig 3F). Lastly, we measured apoptosis through annexin-V and 7-AAD staining (Fig 3H and 3I). No significant changes were observed across all genotypes for CD8^+^ T cells (frequency of CD8^+^Ann-V^+^ WT:11.79% ± 0.87, huCD3ε^HET^:10.60% ± 0.29, huCD3ε^HOM^:10.81% ± 1.05). However, huCD3ε^HOM^ CD4^+^ T cells displayed higher apoptosis compared to those from WT mice (frequency of CD4^+^Ann-V^+^ WT:12.94% ± 1.06, huCD3ε^HET^:13.82% ± 0.40, huCD3ε^HOM^:20.85% ± 2.01).

Taken together these results show that T cells in huCD3ε^HET^ mice retain a normal phenotype when compared to cells from WT mice. In contrast, T cells from huCD3ε^HOM^ mice showed several abnormalities in the periphery. Overall, we identified several alterations in the T cell phenotype of huCD3ε^HOM^ mice, making these mice unsuitable to serve as a surrogate mouse model to test the effect of anti-human CD3 antibodies on biology.

### HuCD3ε^HET^ T cells respond to anti-human CD3 *in vitro* and *in vivo*

**Fig 4:**
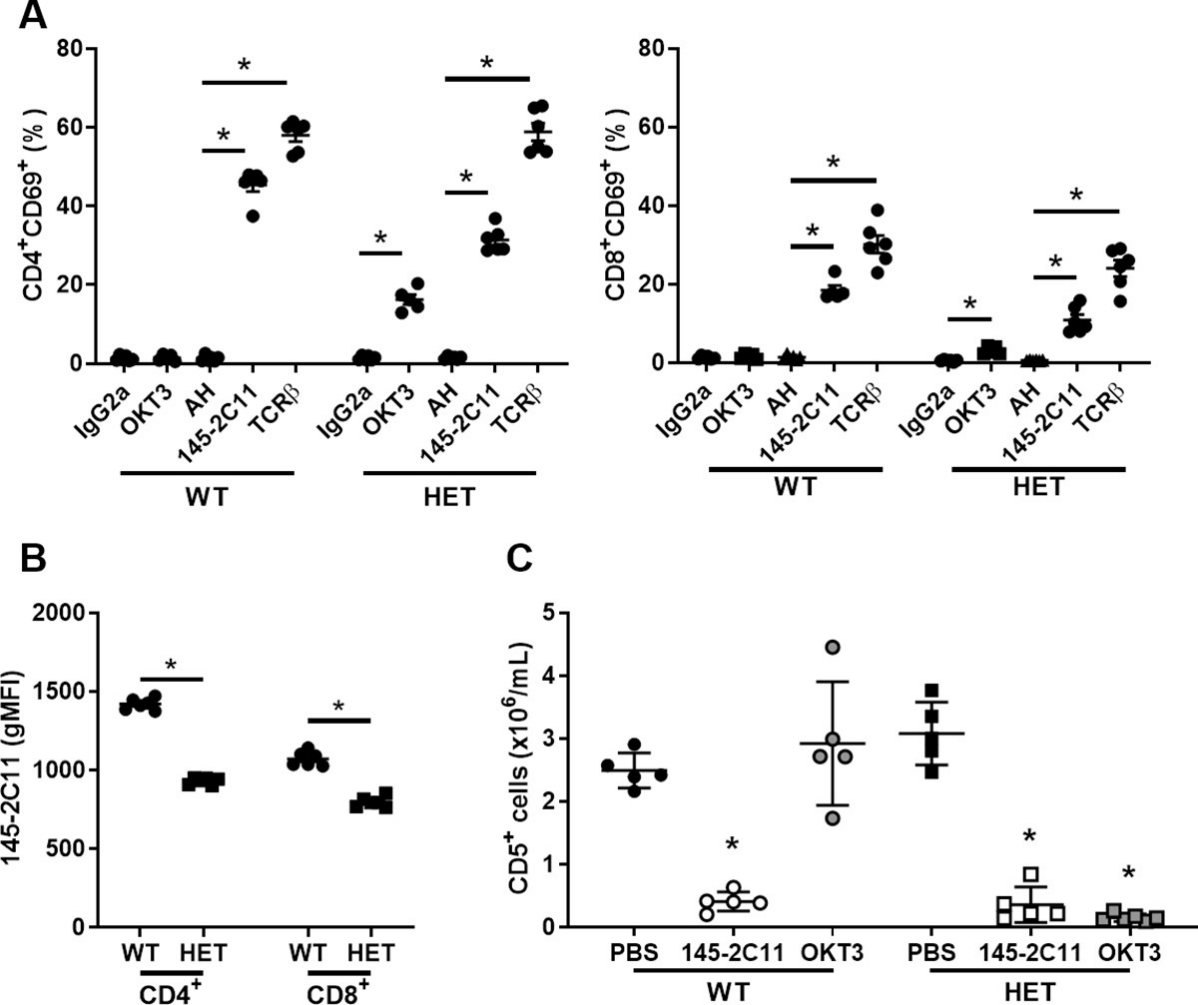
HuCD3ε-expressing T cells respond to anti-human CD3 binding. (A) Splenocytes from WT and huCD3ε^HET^ were cultured *in vitro* for 24hrs with either anti-human (OKT3) or anti-murine (145-2C11) CD3, their respective isotype controls IgG2a or Armenian Hamster (AH), or anti-mouse TCRβ antibodies. CD69 expression was measured by flow cytometry. (B) Geometric mean fluorescence intensity of murine CD3ε as detected by flow cytometry on CD4^+^ and CD8^+^ T cells using 145-2C11 antibody. n = 6, *p<0.05. (C) Mice were administered an i.p. injection of either PBS, anti-mouse CD3 (clone 145-2C11), or anti-human CD3 (clone OKT3). Blood was collected 24hrs post-injection. CD5^+^ T cell numbers in peripheral blood were determined using CBC and flow cytometry. Individual animals are shown with group mean. n = 5, *p<0.05.

To determine the utility of huCD3ε^HET^ mice to study human CD3 biology, we next tested whether introduction of the humanized CD3ε affected *in vitro* and *in vivo* T cell responses. To measure T cell responses *in vitro*, splenocytes from huCD3ε^HET^ and WT were stimulated with plate-bound anti-CD3, anti-TCRβ, or isotype control antibodies for 24 hours, and we compared CD69 surface expression as a measure of activation by flow cytometry. As expected, the positive control anti-TCRβ treatment induced CD69 expression on both huCD3ε^HET^ and WT CD4^+^ and CD8^+^ T cells (Fig 4A). Although T cells from WT mice only upregulated CD69 expression in response to anti-mouse CD3 (145-2C11) activation, T cells from huCD3ε^HET^ mice responded to both 145-2C11 and anti-human CD3 (OKT3) demonstrating that both human and mouse CD3ε are functional (frequency of CD4^+^CD69^+^ WT IgG2a:1.30% ± 0.26, WT OKT3:1.39% ± 0.36, WT AH:1.27% ± 0.30, WT 145-2C11:45.35% ± 1.62, WT TCRβ:57.93 ± 1.54. huCD3ε^HET^ IgG2a:1.40% 0.22, huCD3ε^HET^ OKT3:16.24% ± 1.27, huCD3ε^HET^ AH:1.43% ± 0.16, huCD3ε^HET^ 145-2C11:31.38% ± 1.29, huCD3ε^HET^ TCRβ:58.82% ± 2.23. Frequency of CD8^+^CD69^+^ WT IgG2a:1.28% ± 0.16, WT OKT3:1.51% ± 0.27, WT AH:1.33% ± 0.23, WT 145-2C11:18.44% ± 1.20, WT TCRβ:30.12 ± 2.24. huCD3ε^HET^ IgG2a:0.53% 0.10, huCD3ε^HET^ OKT3:3.07% ± 0.42, huCD3ε^HET^ AH:0.59% ± 0.04, huCD3ε^HET^ 145-2C11:10.86% ± 1.36, huCD3ε^HET^ TCRβ:24.02% ± 2.09). We did observe that CD69 expression levels in huCD3ε^HET^ T cells did not reach the same level induced by 145-2C11 in WT. This could be due to cells dually expressing the murine and humanized-CD3ε genes. In line with these data, the intensity of CD3 staining using 145-2C11 antibody was decreased in huCD3ε^HET^ T cells compared to WT cells (gMFI of 145-2C11 in CD4^+^ T cells WT:1418.67 ± 14.83, huCD3ε^HET^:930.33 ± 8.95. gMFI of 145-2C11 in CD8^+^ T cells WT:1071.33 ± 18.86, huCD3ε^HET^:797.33 ± 13.54) (Fig 4B). Despite this, anti-TCRβ stimulation activated T cells from both genotypes to the same degree suggesting huCD3ε-expression does not lead to aberrant TCR signaling.

Finally, we tested the consequences of injecting anti-CD3 (OKT3 or 145-2C11) antibodies into naïve huCD3ε^HET^ or WT mice (Fig 4C). In accordance with previously reported results in mice and human clinical trials, anti-CD3 antibody injection led to a decrease in T cells numbers from our mice (absolute CD4^+^ T cell number in WT PBS:2.49x10^6^ ± 0.12, WT 145-2C11:0.41x10^6^ ± 0.07, WT OKT3:2.92x10^6^ ± 0.44. Absolute CD8^+^ T cell number in WT PBS:3.08x10^6^ ± 0.22, WT 145-2C11:0.36x10^6^ ± 0.13, WT OKT3:0.15x10^6^ ± 0.02) [8, 30]. Specifically, 145-2C11 antibody injection led to a significant decrease in T cell numbers in the spleen of WT mice. A similar effect was seen in huCD3ε^HET^ mice. Moreover, injection of OKT3 led to decreased T cell numbers only in huCD3ε^HET^ mice. These results demonstrate that both mouse and human CD3ε molecules are expressed and are functional in huCD3ε^HET^ mice.

### HuCD3ε^HET^ mice respond to immunological challenge

**Fig 5:**
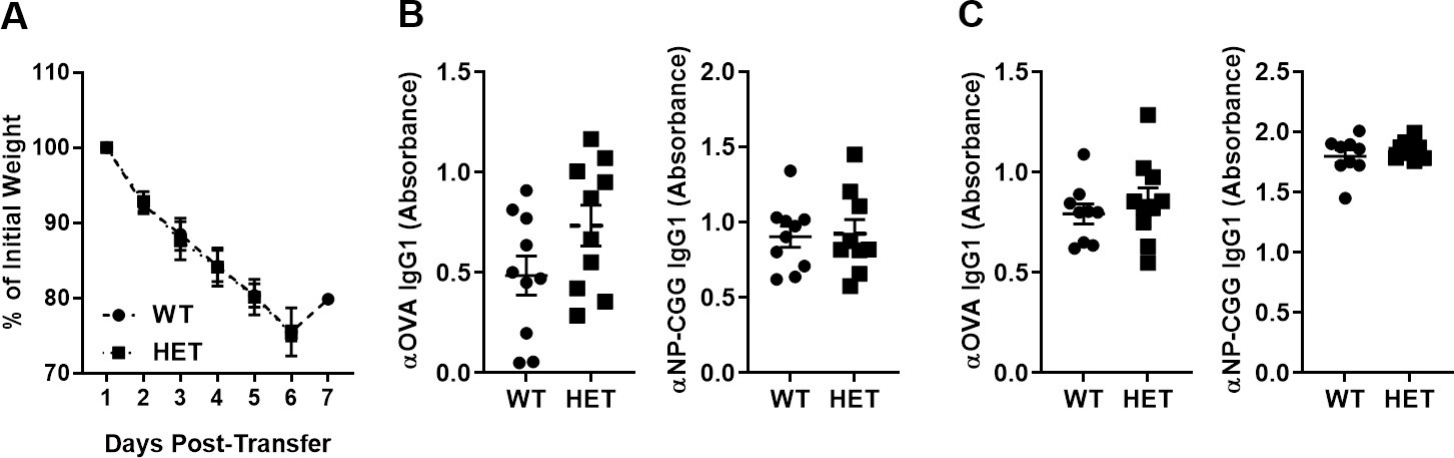
HuCD3ε-bearing T cells develop functional immune responses. (A) Splenocytes from 3–4 month old mice from WT or huCD3ε^HET^ mice were injected i.v. into 8–9 week old, 800 rads irradiated, B6D2 mice and graft-vs-host disease developed *in vivo* after a week post-injection. Percent body weight change is shown (A). n = 3–7, *p<0.05. (B-C) 2–4 month-old mice WT and huCD3ε^HET^ were immunized against ovalbumin or NP-CGG and administered a booster shot on day 14. Antibody titers were measured on day 10-post immunization (primary response) (B) or seven days following booster shot (secondary response) (C) for each antigen. n = 10, *p<0.05.

Since cells responded to anti-CD3 stimulation *in vitro*, we evaluated the capacity of huCD3ε^HET^ T cells to mount functional immune responses *in vivo* using two T cell-dependent models. We first directly tested T cell responses in a T cell-dependent *in vivo* model of graft-vs-host disease [31]. This was done through transfer of huCD3ε^HET^ or WT splenocytes into irradiated allogeneic B6D2 8–9 weeks-old host mice and measuring body weight loss as the disease progressed (Fig 5A). B6D2 host mice which received huCD3ε^HET^ cells lost body weight at the same rate as B6D2 mice which received allogeneic WT cells, demonstrating that huCD3ε^HET^ T cells can be activated similarly to WT T cells (percentage of initial body weight WT Day1:100.00% ± 0.00, Day 2:92.31% ± 0.42, Day 3:88.49% ± 0.81, Day 4:84.26% ± 0.79, Day 5:80.34% ± 0.58, Day 6:75.48% ± 1.44, Day 7:79.86% ± 0.00. Percentage of initial body weight huCD3ε^HET^ Day1:100.00% ± 0.00, Day 2:92.85% ± 0.50, Day 3:87.62% ± 0.96, Day 4:84.12% ± 0.96, Day 5:80.12% ± 0.89, Day 6:75.29% ± 0.57, Day 7: NA). Secondly, we tested the production of antibodies to ovalbumin and NP-CGG which rely on T and B cell interactions for antibody production. We immunized huCD3ε^HET^ and WT mice with OVA and NP-CGG antigens and measured the primary responses by detecting antibody titers in serum 10 days post-immunization (Fig 5B). Antibody titers did not differ between huCD3ε^HET^ and WT mice (absorbance αOVA IgG1 WT:0.49 ± 0.10, huCD3ε^HET^:0.73 ± 0.10. Absorbance αNP-CGG IgG1 WT:0.90 ± 0.07, huCD3ε^HET^:0.93 ± 0.09). We followed up by challenging immunized mice with a boost on day 14 post-immunization and similarly measured antibody titers in serum and again found no significant differences between huCD3ε^HET^ and WT mice (absorbance αOVA IgG1 WT:0.79 ± 0.05, huCD3ε^HET^:0.86 ± 0.07. Absorbance αNP-CGG IgG1 WT:1.80 ± 0.05, huCD3ε^HET^:1.86 ± 0.02) (Fig 5C).

Taking the results from both *in vivo* models together, we show that huCD3ε^HET^ T cell responses are unimpaired and T cells are able to perform their effector functions making this model suitable to further test pre-clinically the effect of anti-human CD3 therapy in different models of disease in the C57BL/6 background.

## Discussion

There is interest in understanding how anti-CD3 therapy may be used in autoimmune and oncology patients. For example, teplizumab and otelixizumab have both been used in clinical trials for Type 1 diabetes and efficacy was detected up to seven or four years following short-term treatment, respectively [9, 13, 30, 32–34]. Similarly, it has been posited that anti-CD3 bi-specific antibodies may be used to stimulate T cell responses against tumors [6, 35]. A major hurdle to studying anti-CD3 therapy in mice relies in species specificity where anti-human CD3 antibodies do not react with murine CD3 and the genetically modified mouse models that have been developed each have specific caveats.

The mice in the current study were generated by replacing the murine CD3ε exon with the corresponding humanized CD3ε exon. Our aim was to include the binding epitope that anti-human CD3 antibodies recognize but also keep the native interaction of the humanized CD3ε with murine CD3 subunits CD3δ and CD3γ. We did this by replacing the homologous murine exon 5 with the human exon 6 and mutating the Arginine sequence at the C terminal end of the human exon 6 to a Lysine sequence [26, 27]. By using this approach, we observed anti-human CD3 antibodies bound to huCD3ε-expressing cells, but not WT. To our surprise (and despite these efforts to minimize the effect of introducing huCD3ε), we observed numerous abnormalities in the huCD3ε^HOM^ mice. Taken together these results suggest the successful integration of the humanized CD3ε gene into murine hosts. Future goals would be to look at the humanized CD3 structure and interactions among its subunits.

Previous reports aimed to address this question by introducing human CD3 into mice [8, 16]. Full-length human CD3ε was introduced as a transgene into NOD mice. Similar to NOD-huCD3ε mice, our huCD3ε^HET^ mice have dual expression of the murine and the human CD3ε-exon which allows the ability to study both anti-human and anti-murine CD3 responses [16]. An advantage to using our huCD3ε^HET^ over NOD-huCD3ε lies in their C57BL/6 background which can be crossed to various antigen specific models such as PMEL-1 or OT-I/II and used for modeling antigen specific responses and phenotype following challenging with anti-human CD3 therapy. Further, huCD3ε^HET^ mice develop normally, are immune competent, and do not develop disease, unlike NOD-huCD3ε mice which develop diabetes, allowing us to test anti-human CD3 treatment in the context of animal models for both autoimmune and malignant diseases.

The most recent mouse model described, which integrates the human CD3 complex through expression of CD3ε, CD3δ, and CD3γ was shown to be a robust *in vivo* model to study anti-human CD3 therapy [17]. However, human *CD3 EDG* mice show discreet changes in CD3 frequency and CD4:CD8 ratio vs. WT mice, whereas the huCD3ε^HET^ mice described here are indistinguishable from WT mice. These differences result in changes in the final immune response. In fact, the authors observed subtle changes in the level of IgG1 and IgE antibodies produced following secondary exposure to a specific antigen between WT and human *CD3 EDG* mice. We did not observe any changes in antibody titers, following immunization against T cell-dependent antigens ovalbumin or NP-CGG between huCD3ε^HET^ vs WT mice. Lastly, the authors observed differences in thymus weights, which taken together with the changes in CD4:CD8 ratios, would suggest human *CD3 EDG* thymocyte selection is somewhat impaired. This could lead to aberrant TCR diversity and therefore, impaired antigen detection by T cells. Here we show that huCD3ε^HOM^ mice similarly showed aberrant thymocyte development which led to impaired T cell numbers, phenotype and survival in the periphery. In this regard, huCD3ε^HOM^ mice are reminiscent of a previously developed model where murine T cells which expressed high levels of human CD3 gene also showed truncated thymocyte development [15]. These differences rendered huCD3ε^HOM^ mice inadequate to answer our questions regarding anti-human CD3 therapy, though they may still be of interest to study thymocyte development. However, we have shown that the huCD3ε^HET^ mouse model described here improves upon existing human CD3-expressing mouse models as these retain normal T cell phenotype, proper *in vivo* responses, and respond to anti-human CD3.

A different approach is to engraft the human immune system into immune-deficient mice [18, 19]. As stated earlier, this model has specific limitations in immune cell subsets developing properly—importantly for us, T cells. Reports have circumvented this in part by genetically overexpressing human proteins in mice or through co-transplantation of human fetal liver and autologous CD34^+^ stem cells into immune-deficient mice to reconstitute the human immune system to allow for cells to better develop to varying degrees of success [23–25]. However, obtaining such autologous-matched tissue may be difficult and expertise in mouse surgery are inherent limitation to this model. Further, this approach does not ensure reproducibility between donors, is expensive, time-consuming as mice are analyzed 18–25 weeks following transplantation, and may not recapitulate the same frequencies of T cell subsets *in vivo*. In contrast to human-engrafted immune-deficient mice, by replacing a single murine CD3ε-exon with the corresponding humanized CD3ε-exon, the huCD3ε^HET^ mice show normal thymus, thymocyte development and retain normal T cell numbers and phenotype in the periphery. This allows the normal development of the murine immune system. We observed huCD3ε^HET^ T cells retained comparable naïve-to-memory ratio, activation and apoptosis levels to their WT counterparts. We did not observe any cellular aberrations in huCD3ε^HET^ mice. We also observed that huCD3-expressing cells were depleted upon injection with anti-human CD3 antibodies *in vivo* as observed in human clinical trials where T cell numbers in blood is used a common PD marker for engagement of anti-CD3 with its target. Further, huCD3ε^HET^ T cells responded to anti-human CD3 by increasing CD69 surface expression. All these results suggest that huCD3ε^HET^ mice are a suitable tool to study biology following treatment with anti-CD3 antibodies.

Lastly, we challenged and measured the immune response of huCD3ε^HET^ mice through a graft-vs-host model that relies on T cell effector function [31]. We transferred T cells into allogeneic hosts and found huCD3ε^HET^ T cells were able to induce a wasting disease as measured by body weight loss. Although discreet differences were observed upon *in vitro* activation of huCD3ε^HET^ with 145-2C11 and OKT3, we observed that T cells reach the same level of activation (CD69 expression) when stimulated with anti-TCRβ, suggesting lower activation may be explained by dual expression of the humanized and murine CD3ε-exon. We therefore recognize that signaling through the CD3-complex of huCD3ε^HET^ T cells differ compared to WT T cells. This can be seen in the difference in CD69^+^ T cell frequency following stimulation with anti-CD3 antibodies and discreet changes in CD8^+^ T cell TCR-Vβ usage. Nevertheless, *in vivo* responses to antigen immunizations and graft-vs-host model were not different between huCD3ε^HET^ and WT mice.

Evaluating drug candidates in pre-clinical models is a crucial step in translating bench science to the clinic. As it stands, there are several models that can be used to study the effects of anti-human CD3 treatment, each with their specific advantages and disadvantages. The huCD3ε^HET^ mouse model we describe here may address several of the problems encountered before, as described above. Our mouse model offers the versatility of being able to be backcrossed into several C57BL/6 strains (i.e. PMEL, OT-I, or OT-II) or NOD background to study antigen specific responses as well as classical T cell transfer immunological studies. Future experiments in these mice should look to determine the *in vivo* consequences of anti-CD3 therapy on T cell depletion and endothelial margination, circulation into other organs and/or tissues, modulation of T cell effector functions in disease or non-disease settings as well as (in the case of the huCD3ε^HOM^ mice) better understanding thymocyte development.

## Supporting information

S1 Fig TCR-Vβ profile of huCD3ε-bearing T cells. (A-B) TCR-Vβ profile of 2-month old WT, huCD3e^HET^ and huCD3e^HOM^ CD4^+^ and CD8^+^ T cells. n = 8, *p<0.05.
(TIF)

S2 Fig T cell numbers of huCD3ε^HOM^ normalize in aged mice. (A-B) Absolute overall, naïve and memory CD4^+^ (A) and CD8^+^ (B) T cell numbers from splenocytes of WT, huCD3ε^HET^ and huCD3ε^HOM^ mice at 6, 9, 12 and 17 months of age. n = 3–14, *p<0.05.
(TIF)
