# Supplementary figures and images for: A humanized CD3ε-knock-in mouse model for pre-clinical testing of anti-human CD3 therapy
Source: PLoS One. 2021 Feb 17;16(2):e0245917. doi: 10.1371/journal.pone.0245917 (PMC7888618; doi:10.1371/journal.pone.0245917)

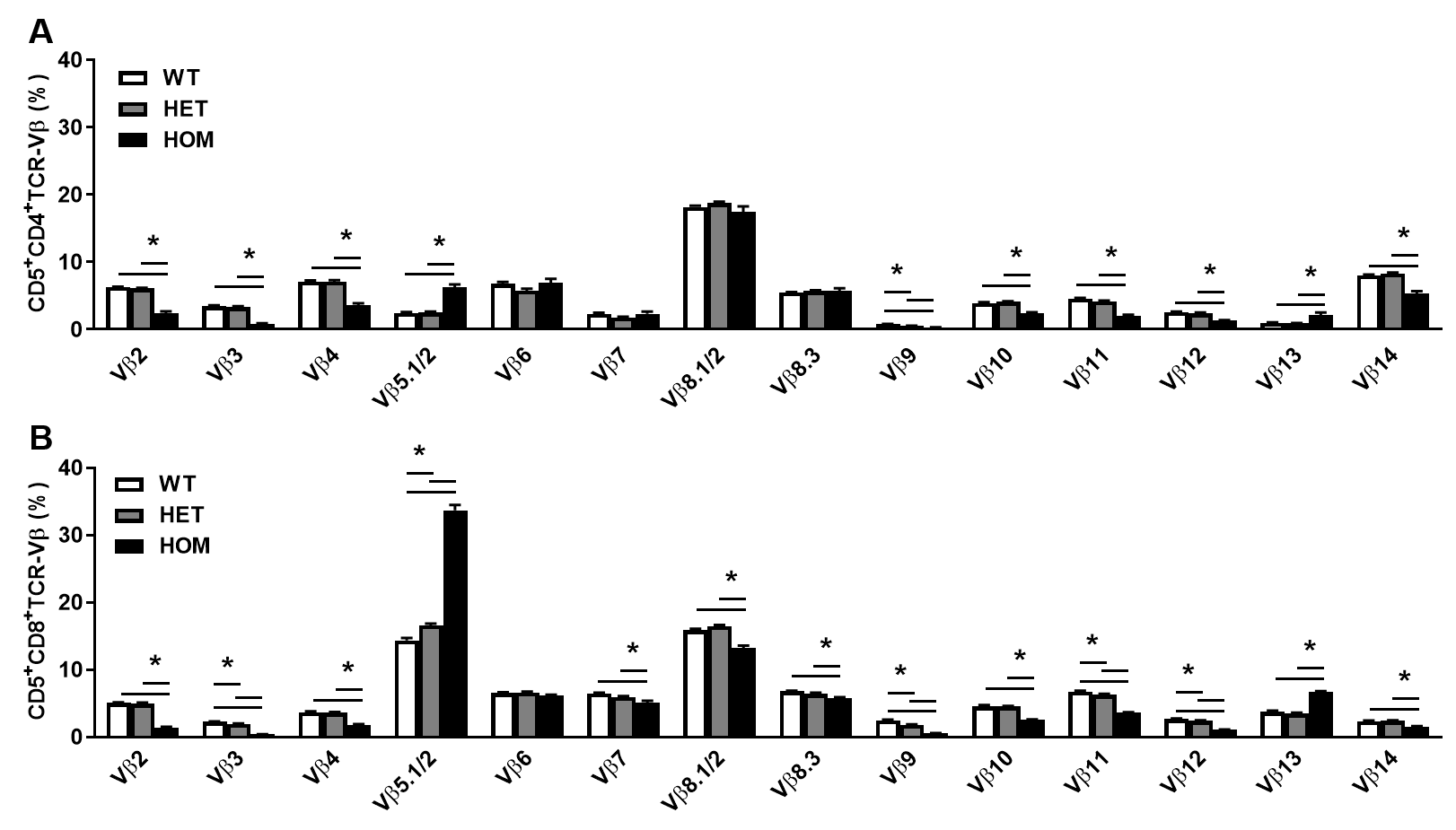

Supplement: S1 Fig — (A-B) TCR-Vβ profile of 2-month old WT, huCD3eHET and huCD3eHOM CD4+ and CD8+ T cells. n = 8, *p<0.05. (TIF) [file pone.0245917.s001.tif]

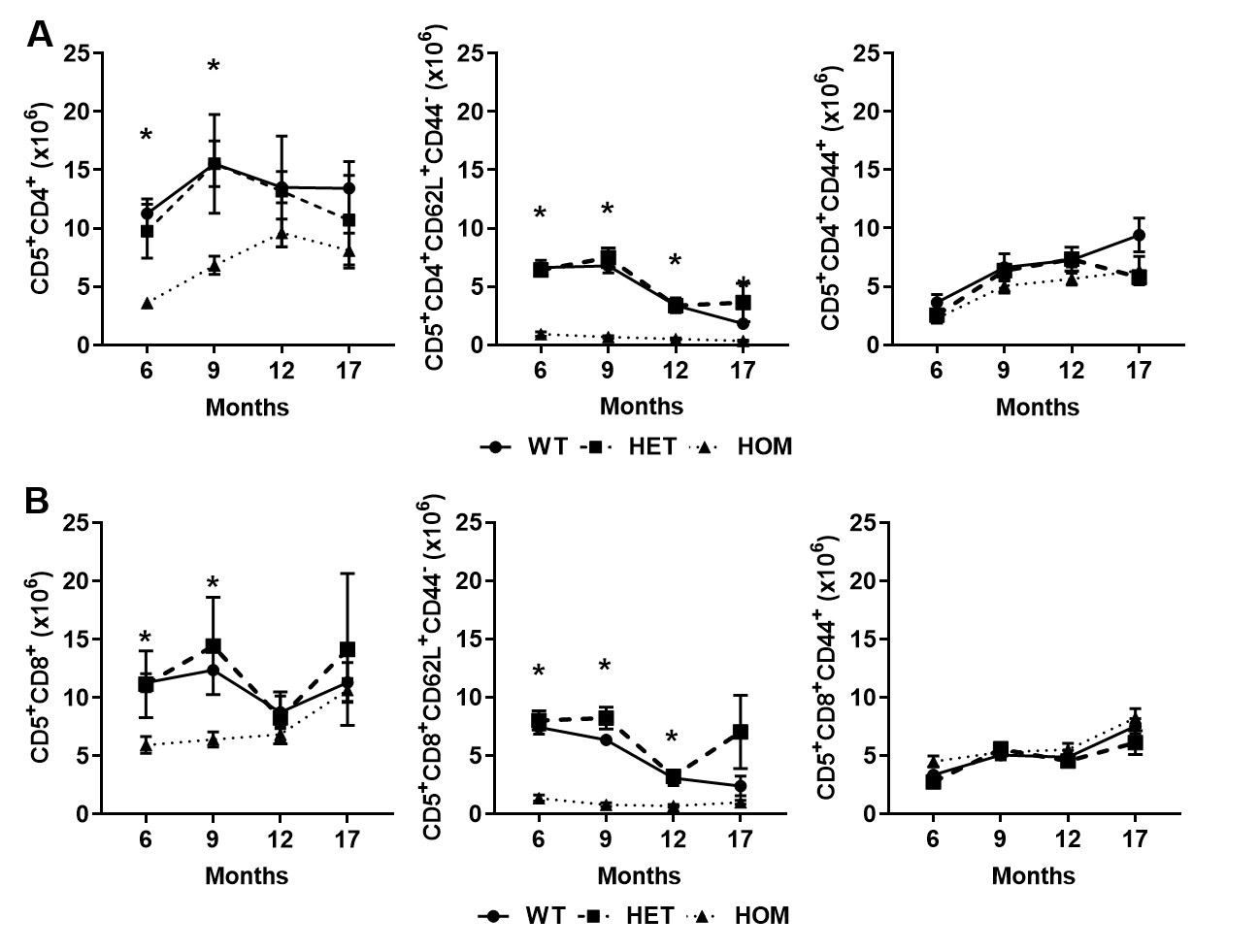

Supplement: S2 Fig — (A-B) Absolute overall, naïve and memory CD4+ (A) and CD8+ (B) T cell numbers from splenocytes of WT, huCD3εHET and huCD3εHOM mice at 6, 9, 12 and 17 months of age. n = 3–14, *p<0.05. (TIF) [file pone.0245917.s002.tif]
